# Plasma activated water effects on behavior, performance, carcass quality, biochemical changes, and histopathological alterations in quail

**DOI:** 10.1186/s12917-024-04231-4

**Published:** 2024-09-04

**Authors:** Usama T. Mahmoud, Ghada Abd El-Reda, Fatma Abo Zakaib Ali, Manal A.M. Mahmoud, Sherief M.S Abd-Allah, F. M. El-Hossary, Nasser S. Abou Khalil

**Affiliations:** 1 Department of Animal, poultry and aquatic life behavior and management, Faculty of Veterinary Medicine, Assiut University, Assiut, 71526 Egypt; 2 Physics Department, Faculty of Science, Assiut University, Assiut, 71516 Egypt; 3 Department of Pathology and Clinical Pathology, Faculty of Veterinary Medicine, Sohag University, Sohag, 82524 Egypt; 4 Department of Animal Hygiene and Environmental Pollution, Faculty of Veterinary Medicine, Assiut University, Assiut, 71526 Egypt; 5 Department of Food Hygiene, Safety and Technology (Meat Hygiene, Safety and Technology), Faculty of Veterinary Medicine, Assiut University, Assiut, 71526 Egypt; 6 Physics Department, Faculty of Science, Sohag University, Sohag, 82524 Egypt; 7 Department of Medical Physiology, Faculty of Medicine, Assiut University, Assiut, 71515 Egypt; 8 Department of Animal Physiology and Biochemistry, Faculty of Veterinary Medicine, Badr University, Assiut, Egypt

**Keywords:** Plasma activated water, Quail, Behaviour, Performance, Blood biochemical, Histopathology

## Abstract

**Background:**

Plasma-activated water (PAW) is an innovative promising technology which could be applied to improve poultry health. The current study investigated the effects of drinking water supply with PAW on quail behaviour, performance, biochemical parameters, carcass quality, intestinal microbial populations, and internal organs histopathology. A total of 54 twenty-one-day-old Japanese quail chicks were randomly allotted to three treatments provided with PAW at doses 0, 1 ml (PAW-1), and 2 ml (PAW-2) per one litter drinking water. Each treatment contained 6 replicates (3 birds/ cage; one male and two females).

**Results:**

The results clarified that there were no significant *(P > 0.05)* changes in behaviour, and performance. For the biochemical indicators, the PAW-1 group showed significantly higher serum H_2_O_2_, total protein and globulin levels compared with the other groups *(P = 0.015*,* < 0.001*,* and 0.019;* respectively*)*. PAW groups had significantly lower serum creatinine and urea levels than the control *(P = 0.003)*. For the carcass quality, the internal organs relative weight between different treatments was not changed. In contrast, there was a significant increase in the meat colour, taste, and overall acceptance scores in PAW groups compared with the control one *(P = 0.013*,* 0.001*,* and < 0.001;* respectively*)*. For the intestinal microbial population, *lactobacilli count* was significantly higher in PAW-2 compared with the control group *(P = 0.014)*, while there were no changes in the total bacterial count between different treatment groups. Moreover, mild histological changes were recorded in the intestine, liver, and spleen of PAW groups especially PAW-2 compared with the control one.

**Conclusions:**

PAW offered benefits, such as reducing creatine and urea levels, improving meat characteristics, and increasing lactobacilli count, all of which are crucial for sustainable quail farming. Therefore, further research is needed.

## Background

Quail production is one of the poultry industry’s expanding segments. Consumers greatly enjoy the diversity and good nutritional quality that quail meat offers to the human diet. Compared to other poultry species, quail offers several advantages, such as rapid growth, high productivity, early laying onset, high rates of reproduction, low feed intake, low investment, and disease resistance [1–4]. The significant increase in the rates of meat and egg production seen in quail farming can be attributed to advancements in genetics, better management practices, environment, and nutrition, as well as the growth of research employing various feed ingredients to satisfy production demands [5]. A societal issue with significant economic implications, antibiotic-resistant bacteria are becoming more prevalent in quail [6, 7]. The discovery of a naturally occurring chemical as an antibiotic substitute may contribute to better animal and human wellbeing. One of the established feeding strategies is the use of acidic compounds that have antibacterial activity to improve the performance and health status of quail and control the antibiotic resistance problem [8].

PAW is a relatively new and innovative technology that has gained attention in various industries, including the poultry industry. PAW is created by subjecting water to a plasma discharge, that generates chemically reactive species such as reactive oxygen species and reactive nitrogen species, which makes PAW acidic [9–12].These species can have various antimicrobial and disinfectant properties, making PAW potentially useful in poultry farming and processing [13–15]. The PAW antibacterial modes of action were reviewed by Zhao et al. [16] and subdivided into 4 main modes of action as fellow: First, primary and secondary reactive oxygen and nitrogen species (RONS) are generated in PAW, leading to reduced pH, increased ORP, and electrical conductivity, which cause physical stress on microbial cells. Second, these RONS induce oxidative stress, damaging the peptidoglycan structure of the cell wall and initiating lipid and protein peroxidation on the cell membrane. This results in compromised membrane integrity, shrinkage, distortion, and formation of cracks or pores, followed by depolarization of cell membrane potential. Third, RONS can be transported into the cell through these pores, leading to an accumulation of intracellular RONS and a reduction in intracellular pH due to proton influx. Fourth, the intracellular RONS oxidizes internal components such as DNA, proteins, lipids, and carbohydrates, resulting in DNA fragmentation and protein oxidation, with these oxidized components potentially exiting the cell through the membrane pores [16]. PAW has been shown to inactivate Escherichia coli and Staphylococcus aureus in chicken muscle, rough skin, and smooth skin [13]. It also effectively inactivates Pseudomonas deceptionensis CM2 on the surface of raw chicken breasts [14]. Additionally, due to its antiviral and immunostimulant activities, PAW has been recommended as a promising strategy for the preparation of inactivated vaccines for Newcastle disease [15].

Several studies have shown that PAW is non-toxic and safe for use in animals including healthy CD1 mice [17], immuno-deficient nude mice [18], rabbits [19], and fish [20]. PAW is a promising technology for the poultry industry, but its safety has not been assessed before in different poultry species. Therefore, this pioneering study aimed to assess the impact of PAW oral administration on quail behavior, performance, biochemical parameters, carcass quality, intestinal microbial populations, and internal organ histopathology. We hypothesized that PAW’s low pH and high content of RONS would enhance beneficial microbial populations and intestinal epithelium morphology, thereby improving nutrient absorption, performance, and meat quality. This study offers significant insights into PAW as a unique technique for increasing quail health and productivity. As an environmentally friendly solution, PAW has the potential to improve poultry management and advance sustainable quail farming techniques.

## Results

### The PAW effects on behaviour

Non-significant change in quail behavioral activities (feeding, drinking, standing, sitting, walking, preening, stretching, shaking, feather pecking, and wall pecking) were observed due to PAW treatment *(P > 0.05)* (Table 1).

**Table 1:** The PAW effects on the behavioral activities of quails

Behavioral activities	Treatment groups					
	Control	PAW-1	PAW-2	SEM	F	*P* Value
**Feeding%**	4.86	3.59	5.67	0.86	0.899	0.438
**Drinking%**	5.86	4.86	5.58	0.83	0.295	0.751
**Stand%**	15.12	16.90	14.70	1.74	0.290	0.754
**Sit%**	50.23	48.15	46.98	2.72	0.269	0.770
**Walk%**	10.108	12.384	12.939	1.554	0.723	0.509
**Preening%**	9.182	10.880	9.580	0.991	0.602	0.566
**Stretching%**	1.08	0.93	1.52	0.23	1.029	0.392
**Shaking%**	0.39	0.23	0.15	0.13	0.641	0.547
**Groom%**	10.65	12.04	11.25	1.05	0.350	0.713
**Feather pecking%**	0.77	0.81	0.61	0.33	0.060	0.942
**Wall pecking%**	2.39	1.27	2.27	0.58	0.803	0.475

Behavioral activities of quails (Percentage of certain activity to the total number of activities/cage). (*n* = 6 replicates/group; 18 birds/ group)

### The PAW effects on performance

The results indicate that there were no significant differences (*P* > 0.05) in performance indicators including body weight, body weight gain, feed intake and feed conversion ratio among the different treatments (Table 2).

**Table 2:** The PAW effects on the quails’ performance

**Treatments**	Body weight at age (day)/ g				Body weight gain/g				Feed intake/kg				Feed conversion ratio (g feed/g gain)			
	21	28	35	42	W1	W2	W3	Total	W1	W2	W3	Total	W1	W2	W3	Total
**Control**	71.67	140.06	198.28	244.67	68.39	58.22	46.39	173.00	148.06	136.89	175.11	460.06	2.17	2.37	3.90	2.67
**PAW-1**	71.33	144.50	195.08	242.33	73.17	50.58	47.25	171.00	155.42	129.21	169.83	454.46	2.13	2.57	3.68	2.67
**PAW-2**	69.78	147.89	206.22	253.78	78.11	58.33	47.56	184.00	156.78	134.94	171.00	462.72	2.02	2.32	3.79	2.53
**SEM**	1.84	3.07	3.31	5.98	2.49	2.02	3.86	5.58	4.47	2.45	4.41	7.19	0.07	0.10	0.36	0.08
**F**	0.183	1.164	1.676	0.573	2.619	3.326	0.019	0.885	0.863	2.000	0.326	0.211	0.782	1.272	0.073	0.557
**P value**	0.836	0.351	0.236	0.581	0.122	0.078	0.982	0.443	0.451	0.186	0.729	0.814	0.484	0.322	0.930	0.590

*n* = 6 replicates/group (18 birds/ group; one male and 2 females per cage)

**W1**: First week of experiment; **W2**: Second week of experiment; **W3**: Third week of experiment

### The PAW effects on biochemical parameters

The results in Table 3 indicate that serum H_2_O_2_, total protein, and globulin levels were significantly higher in the PAW-1 group compared to the control group *(P = 0.015*,* < 0.001*,* and 0.019*; respectively). The inclusion of PAW-1 and PAW-2 resulted in a significant reduction in serum creatinine and urea levels compared to the control group *(P = 0.003)*. However, levels of Total antioxidant capacity, albumin, albumin/ globulin ratio (A/G), alanine aminotransferase, and aspartate aminotransferase remained unchanged.

**Table 3:** The PAW effects on the levels of serum biochemical parameters in quails

Parameters	Treatment groups					
	Control	PAW-1	PAW-2	SEM	F	*P* value
H_2_O_2_ (mM/L)	0.51^b^	0.96^a^	0.60^b^	0.09	6.148	0.015
TAC (mM/L)	0.17	0.21	0.13	0.04	1.042	0.383
Albumin (g/dL)	1.93	2.60	2.45	0.19	3.606	0.059
Total protein (g/dL)	3.02^b^	5.45^a^	3.78^b^	0.27	21.321	< 0.001
Globulin (g/dL)	1.09^b^	2.85^a^	1.33^ab^	0.42	5.194	0.019
A/G ratio	1.85	1.42	1.91	0.34	0.627	0.548
ALT (U/L)	9.17	7.17	8.17	1.67	0.356	0.707
AST (U/L)	10.00	10.17	11.33	1.86	0.152	0.861
Creatinine (mg/dL)	0.69^a^	0.23^b^	0.29^b^	0.08	9.997	0.003
Urea (mg/dL)	22.55^a^	12.43^b^	14.07^b^	1.71	10.048	0.003

*n* = 6 replicates/group (18 birds/ group; one male and 2 females per cage); for blood biochemical measures only 6 birds/ group (3males and 3 females) were used. **TAC**: Total antioxidant capacity; **A/G**: albumin/ globulin ratio; **ALT**: alanine aminotransferase; **AST**: aspartate aminotransferase

^a−b^ Means with different superscripts in the same row indicate significant differences at *P* < 0.05 (general linear model followed by Tukey post-hoc test)

### The PAW effects on carcass quality

The outcomes presented in Table 4 demonstrate that the addition of PAW to drinking water had no significant impact *(P > 0.05)* on the relative weights of the empty carcass, gizzard, spleen, heart, and liver. Carcass characteristic measurements (Table 4) revealed that water-holding capacity, pH, juiciness, chewiness, and flavor were similar across all treatments. Values for color in the PAW-2 group were significantly higher than the control group *(P = 0.013)*. Values of taste, and overall acceptability in birds provided with PAW-1 and PAW-2 were significantly *(P = 0.001*,* and < 0.001; respectively)* higher than those in the control group.

**Table 4:** The PAW effects on carcass quality of quails

Parameters	Treatment groups					
	Control	PAW-1	PAW-2	SEM	F	*P* value
Heart %	1.03	0.96	1.21	0.09	1.960	0.175
Liver %	1.98	2.20	1.90	0.31	0.258	0.776
Spleen%	0.05	0.06	0.06	0.01	0.784	0.475
Gizzard%	1.81	1.66	1.86	0.08	1.649	0.225
Empty carcass%	69.25	68.24	72.20	2.46	0.696	0.514
Water-holding capacity	62.01	63.19	63.20	1.61	0.181	0.836
PH	5.868	6.102	6.053	0.159	0.608	0.550
Color	6.50^b^	7.20^ab^	7.48^a^	0.23	4.959	0.013
Taste	6.69^b^	7.34^a^	7.47^a^	0.14	8.322	0.001
Juiciness	7.56	7.89	7.97	0.15	2.268	0.119
Chewiness	7.42	7.93	7.81	0.17	2.333	0.113
Flavor	7.00	7.39	7.39	0.17	1.699	0.199
Overall acceptance	7.03^b^	7.55^a^	7.62^a^	0.09	11.521	< 0.001

*n* = 6 replicates/group (18 birds/ group; one male and 2 females per cage); for carcass quality only 6 birds/ group (3males and 3 females) were used

^a−b^ Means with different superscripts in the same row indicate significant differences at *P* < 0.05 (general linear model followed by Tukey post-hoc test)

### The PAW effects on intestinal microbial populations

**Fig. 1:**
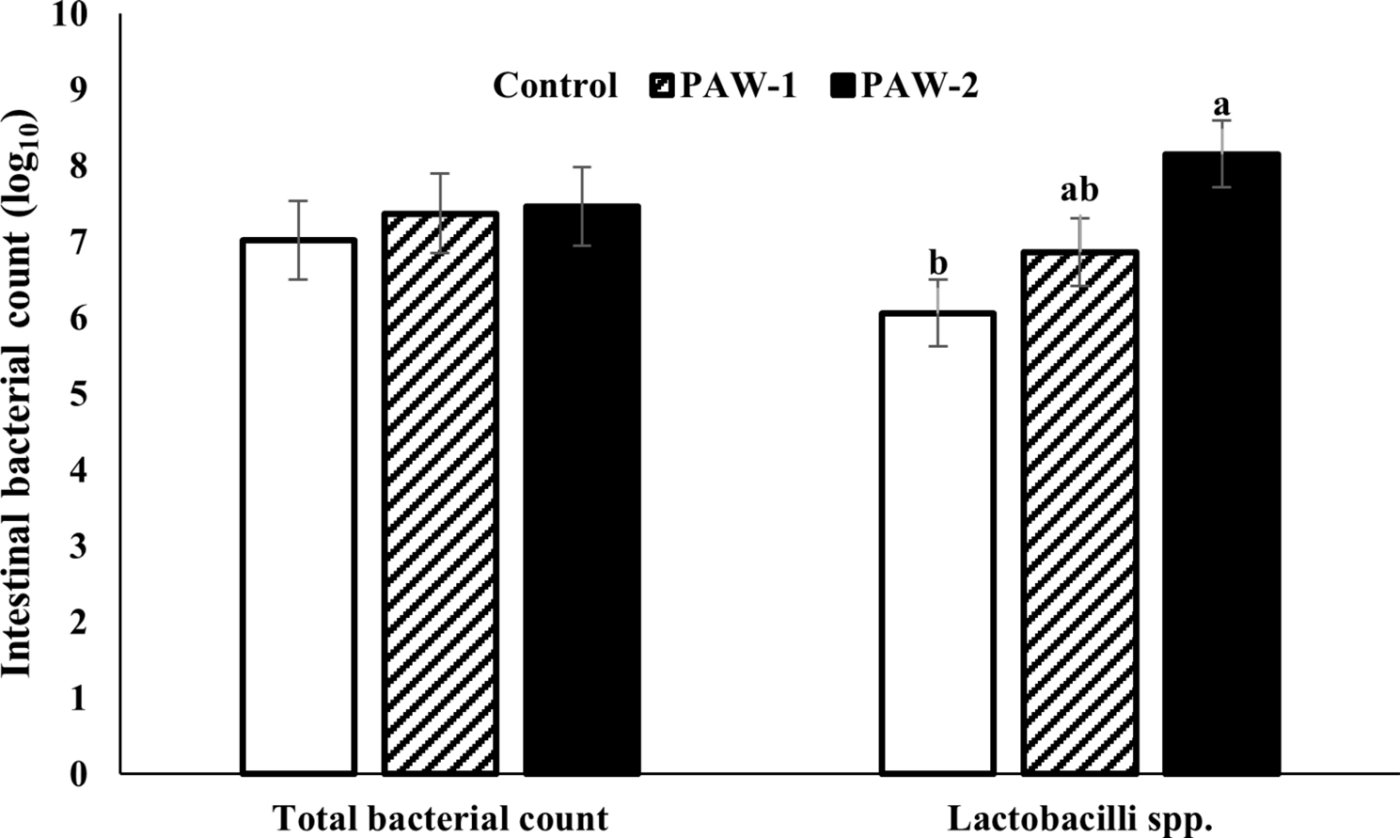
The effects of PAW on intestinal microbial counts in quail. Data are expressed as means ± standard errors. ^a−b^ Different superscripts above different columns for each parameter indicate significant differences (*P* < 0.05)

The data depicted in Fig. 1. revealed that the total bacterial count was not significantly different between treatments *(P > 0.05)*. However, PAW-2 exhibited a significantly higher *Lactobacilli* count than the control group *(P = 0.014)*.

### The PAW effects on internal organs histopathology

**Fig. 2:**
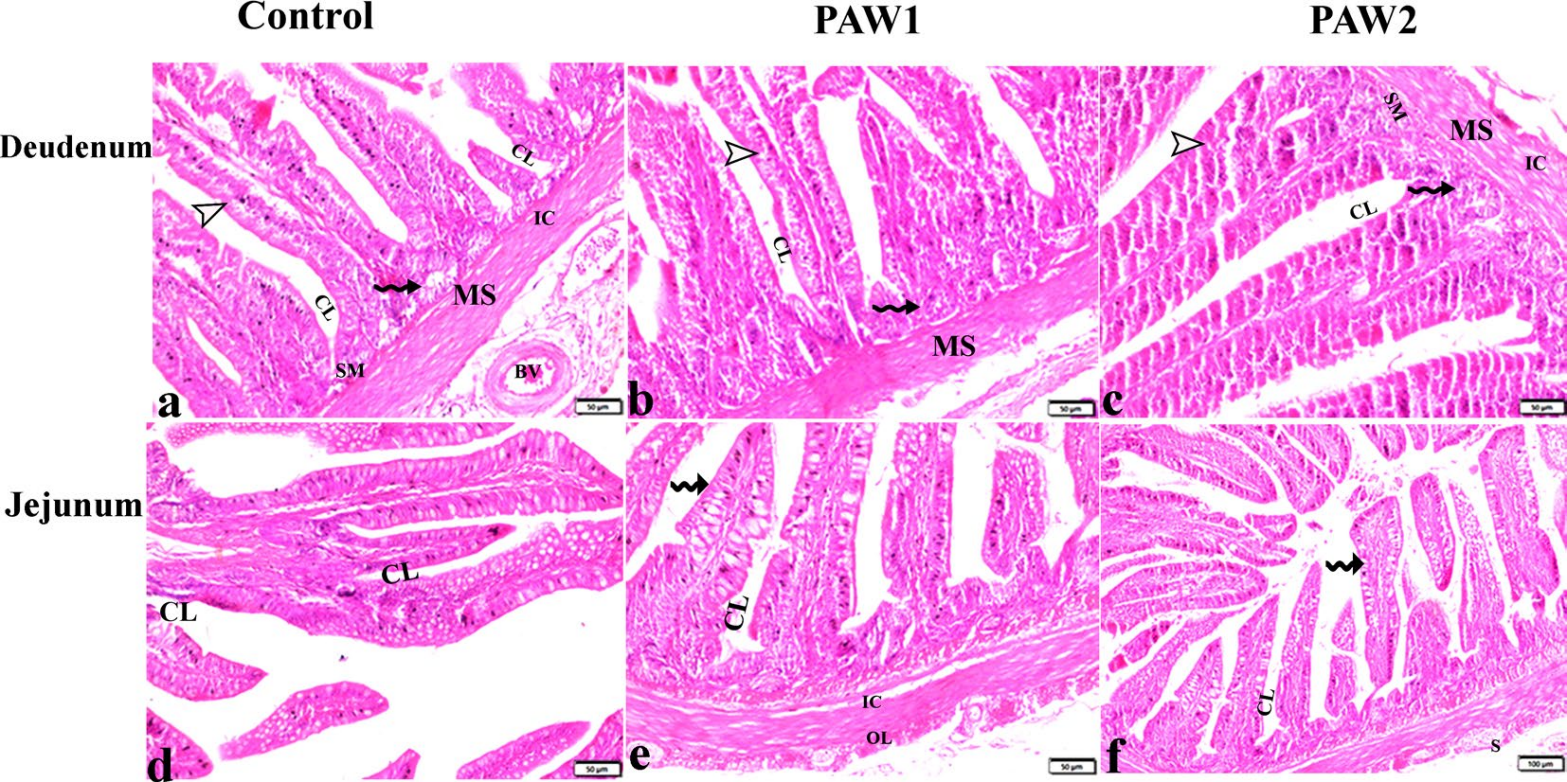
The effects of PAW on duodenum and jejunum histopathological alterations in quail. (**a**-**c**): duodenum tissue sections from experimental groups. villi (V), mucosa (M), submucosa (SM), muscularis (MS), serosa (S) and blood vessel (Bv), inner circular (IC) and the outer longitudinal (OL) muscularis externa, simple columnar epithelial cells with goblet cells (arrowheads), crypts of Lieberkühn (CL), glands of Lieberkühn lined with Paneth cells (zigzag arrows). (**d**-**f**): jejunum tissue sections from experimental groups. villi (V), simple columnar epithelial cells with goblet cells (Zigzag arros), crypts of Lieberkuhn (CL), glands of Lieberkuhn (GL), muscularis mucosa (MM), muscularis externa; inner circular muscle (IC), outer longitudinal muscle (OL), serosa (S). There is no structural difference between the groups

**Fig. 3:**
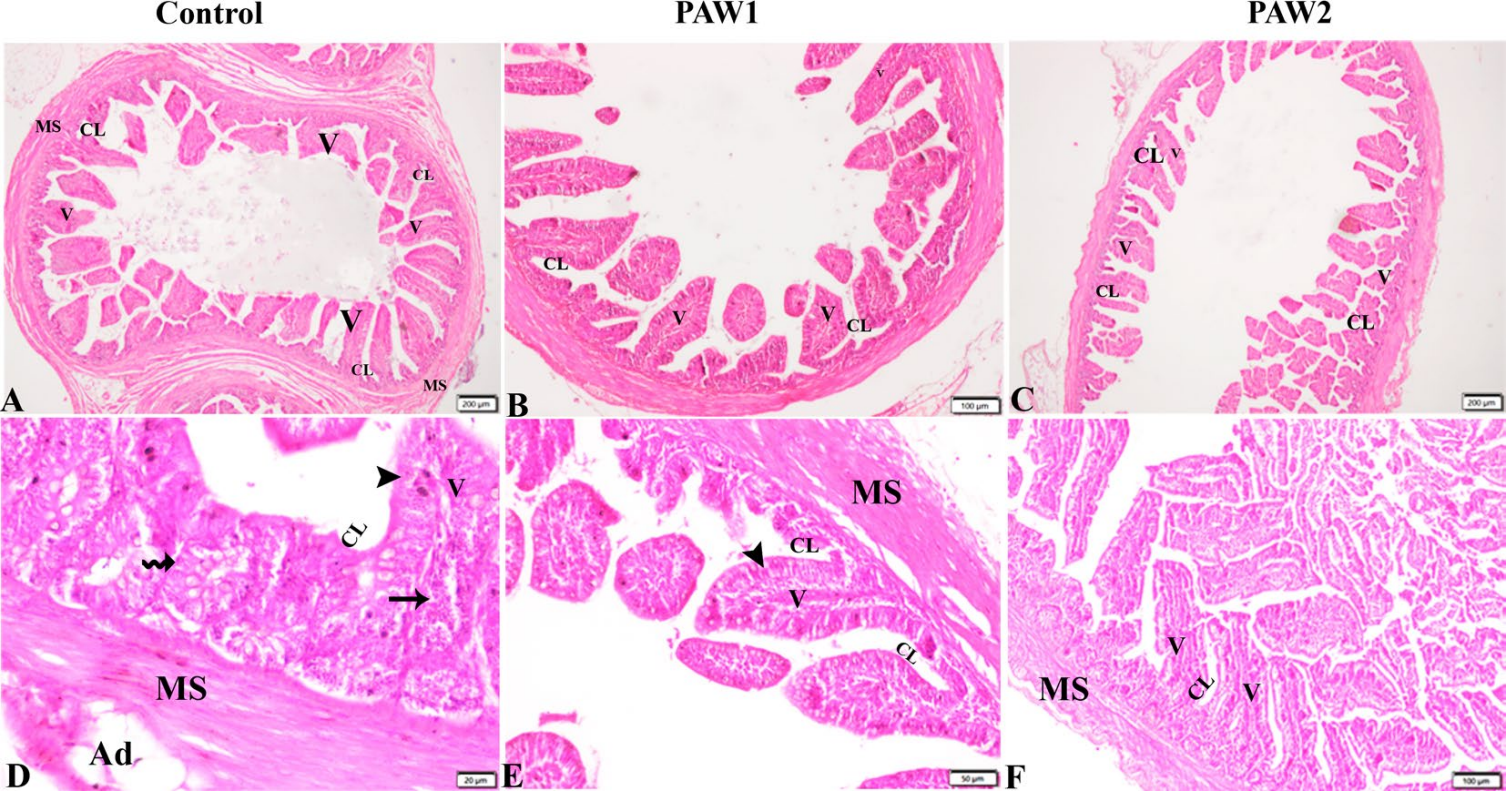
The effects of PAW on ileum histopathological alterations in quail. (**a**-**f**): ileum tissue sections from experimental groups. Control (A magnified in D), PAW1 (B magnified in E), PAW2 (C magnified in F): Villi(V), simple columnar epithelia with goblet cells (arrowheads), the crypts of Lieberkuhn (CL), Glands of Lieberkuhn (Zigzag arrow), muscularis externa (MS). Also shown lacteal (arrow) and adipose tissue (Ad). There is no structural difference between the groups except for a mild decrease in the thickness of mucosal villi from PAW2-treated groups in ileum sections

The PAW effects on intestinal morphometry and histopathology were presented in Table 5; Figs. 2 and 3. indicate that compared to the control group, PAW-treated groups exhibited significantly higher ileum villus height *(P = 0.010)*. However, no significant changes were observed in duodenum and jejunum villus height. PAW-2 showed the highest villus height for both duodenum and jejunum, while duodenum villus height in PAW-1 was significantly lower than in PAW-2. Crypt depth in the jejunum of PAW-1 was significantly reduced *(P = 0.001)* compared to both the control and PAW-2 groups. There were no significant changes *(P > 0.05)* in the villus: crypt ratio among the different treatment groups.

**Table 5:** The PAW effects on the intestinal morphometry of quails

Treatments	Duodenum			Jejunum			Ileum			Average		
	Villus height	Crypt depth	Villus: crypt ratio	Villus height	Crypt depth	Villus: crypt ratio	Villus height	Crypt depth	Villus: crypt ratio	Villus height	Crypt depth	Villus: crypt ratio
**Control**	708.94^ab^	59.44	12.16	140.99	74.22^a^	1.90	256.17^b^	47.50	5.83	389.40	59.13^a^	7.06
**PAW-1**	556.74^b^	45.26	12.14	289.40	22.50^b^	15.59	371.18^a^	51.63	7.22	416.35	41.37^b^	11.29
**PAW-2**	864.66^a^	64.08	13.96	314.69	71.35^a^	4.60	358.47^a^	40.64	11.20	530.60	57.54^a^	10.40
**SEM**	50.96	6.66	1.83	45.66	5.82	3.43	22.33	8.30	1.65	70.79	5.32	1.65
**F**	9.129	2.170	0.323	4.224	24.958	4.458	7.975	0.448	2.867	1.121	3.412	1.827
**P Value**	0.007	0.170	0.732	0.072	0.001	0.065	0.010	0.653	0.109	0.339	0.046	0.178

For intestinal morphometry only 6 birds/ group (3 males and 3 females) were used. Different superscripts within the same column are significantly different at *P* < 0.05 (general linear model followed by Tukey post-hoc test)

**Fig. 4:**
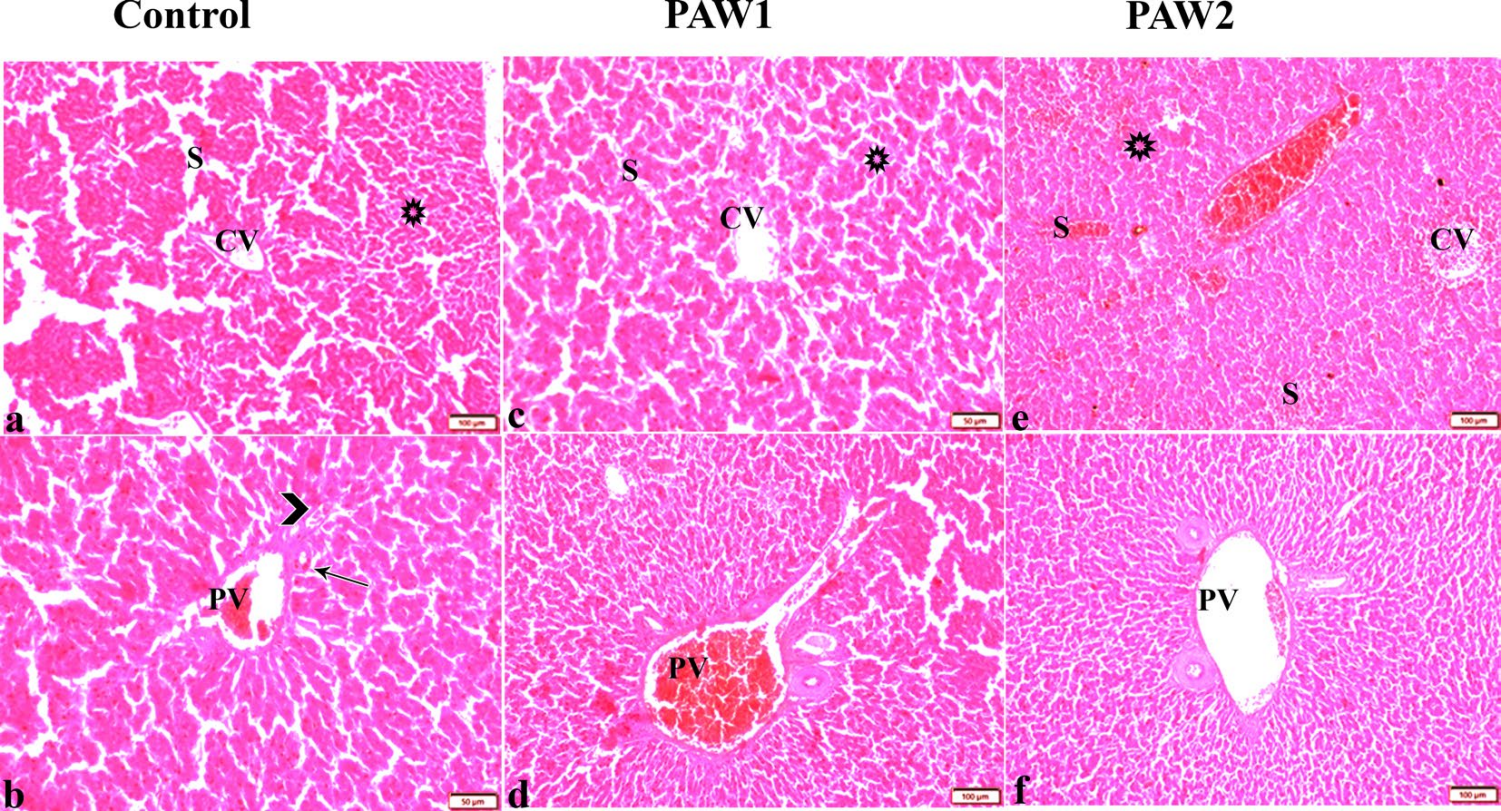
The effects of PAW on liver histopathological alterations in quail. Photomicrograph of liver tissue sections from experimental group stained with HE stains showing: Control: Normal histological hepatic architectures compromising in (a&c): (**a**): normal central veins (CV), normal sinusoids (S), normal hepatocytes arrangement and structure with normal vesicular nucleus (star). (**b**): normal portal triad structure present in; normal portal vein (PV), hepatic artery (arrowhead) and bile duct (arrow). PAW1 (c&d): (**c**): normal central veins (CV), normal sinusoids (S), normal hepatocytes arrangement and structure with normal vesicular nucleus (star). (**d**): congested and dilated portal vein (PV). PAW (e&f): (**e**): congested central vein (CV), sinusoids (S), hepatocellular degeneration (Star). (**f**): dilated portal vein (PV)

The impact of PAW on the histopathological changes in the liver tissue of quails is depicted in Fig. 4. The control groups exhibited an intact hepatic architecture characterized by normal hepatic lobules, central veins, and hepatocyte arrangement, with vesicular nuclei. The portal triad structure, consisting of the portal vein, hepatic artery, and bile duct, appeared normal (Fig. 4a and b). In PAW-1, no significant histological changes were observed in the hepatic tissue, with hepatocellular structure appearing largely normal (Fig. 4c), except for a congested and dilated portal vein (Fig. 4d). However, PAW-2 exhibited various vascular alterations, including congested and dilated central veins and hepatic sinusoids between hepatic cords (Fig. 4e). Additionally, PAW-2 hepatocytes displayed signs of degeneration, and the portal vein appeared distended and congested with blood (Fig. 4f).

**Fig. 5:**
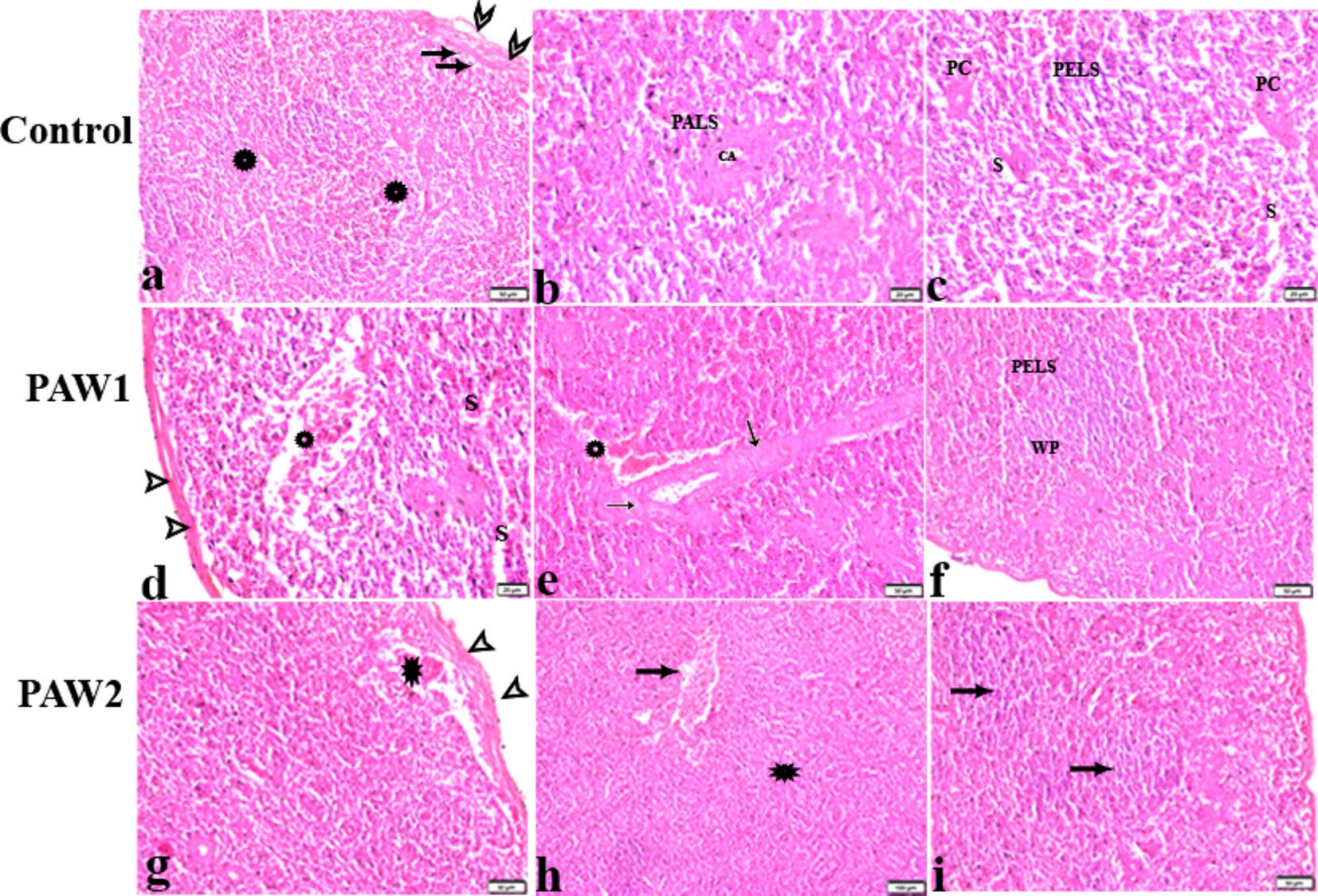
The effects of PAW on spleen histopathological alterations in quail. Histology and general structure of the spleen of from experimental groups: (**a-c**): control normal quail. (**a**) Peritoneal layer covered the capsule (arrowheads) and subcapsular sinuses (arrows), unclear demarcation between white pulp and red pulp (stars). (**B**): PALS were surrounded central artery (CA) and consisted of various white blood cells including lymphocytes, macrophages, reticular cells, and plasma cells. (**C**): Penicilliform capillaries (PC) were encircled by PELS. PELS (lymphatic nodule) were formed mainly of lymphocytes of different sizes, macrophages, reticular cells, and heterophils. The Penicilliform (PC) continued with the blood sinuses (S). (**d-f**): PAW1 showing: (**d**): normal splenic capsule (arrowheads), dilated and congested sinuses (S). splenic venules dilatation and congestion (A&B stars). (**d**): thickening and hyalinization of splenic arterioles (arrows). (**e**): hyperplasia in PELS (WP). (**g-i**): PAW2 showing: (**g**): mild thickening in splenic capsule (arrowheads), dilated and congested sub capsular sinus (star). (**h**): splenic venules dilatated and congested (arrow). Normal lymphoid cellular density in splenic parenchyma (star). (**i**): lymphocytic hyperplasia in PELS (arrows)

The impact of PAW on the histopathological changes in the spleen tissue of quails is illustrated in Fig. 5. Histological examination revealed the stroma and parenchyma of the spleen, characterized by a thin capsule and the absence of trabeculae. Distinct demarcation between white pulp and red pulp was not observed. Central arteries gave rise to penicilliform capillaries, encircled by periellipsoidal lymphatic sheaths (PELS) (Fig. 5a). White pulp comprised periarterial lymphatic sheaths (PALS) and PELS, surrounded by arterioles and ellipsoids, respectively (Fig. 5b). Both PALS and PELS contained various white blood cells. Red pulp consisted of sinuses and cords (Fig. 5c) containing diverse cell types. In the PAW-1 treated group, mild vascular changes were observed, including dilatation and congestion in splenic sinuses, venules, and arterioles, with thickening and hyalinization of arterioles. Additionally, mild cellular changes such as lymphocytic depletion in PALS and hyperplasia in PELS were noted (Fig. 5d and e). The PAW-2 treated group exhibited mild thickening of the splenic capsule and dilated, congested subcapsular sinuses and venules. The lymphoid cellular density in the splenic parenchyma was normal, with mild hyperplasia observed in PELS (Fig. 5g-i).

## Discussion

The drinking water pH is a critical factor for promoting overall bird health and productivity. Recently, several research studies have recommended adding acidifiers such as organic acids due to their low pH and antibacterial activities in poultry drinking water to improve the bird’s health and productivity [21–23]. PAW has the similar advantages therefore the current study suggested it may be beneficial for improving the quail health. To our knowledge, this is the first study to evaluate the effects of PAW on one of the poultry species (quail). Several previous studies have predominantly examined the potential toxicity of plasma-activated liquid and PAW on various animal models. Rabbits subjected to plasma-activated liquid injection into bone marrow for 30 days displayed no adverse effects [19]. Likewise, mice administered PAW over 90 days exhibited no toxicity signs or behavioural abnormalities. Key indicators, such as skin colour, mucous membrane state, piloerection, diarrhoea, weight loss, food intake reduction, locomotor activity, and body posture, remained unaffected. This suggests the absence of adverse effects associated with PAW treatment in the tested mice [17]. However, CD1 female mice consuming PAW ad libitum for 180 days exhibited reduced appetite, vigour, and body weight [24]. Similarly, the current study recorded no changes in quail behaviour in PAW groups in compared with the control group. Quail behaviour can serve as an indicator of health status and well-being [25, 26]. Behavioural responses in quail can be used to evaluate chemical toxicity [24, 27]. Studies have shown that behavioural measures are often more sensitive and persistent than traditional physiological measures; and Japanese quail have been used as a model for avian toxicity tests due to their ease of husbandry and quick reproductive maturity [28].

Body weight and feed intake have significant effects on the health state of quail. High body weight resulted in better performance, including higher egg production and feed efficiency, compared to low body weight [29]. The composition of drinking water can profoundly influence body weight and feed intake in quails, as demonstrated by several studies on different additives such as common salt, moringa leaf solution, aqueous anise seed extract, and liquid probiotics [30–33]. However, there is no specific information available on the effect of PAW composition on quail body weight and feed intake. The current study reported that, quails supplemented with PAW-2 exhibited numerically higher total body weight and body weight gain compared to both the control group and PAW-1, although these differences were not statistically significant. The PAW-2 group exhibited the best feed conversion ratio (2.53), while the other groups recorded a ratio of 2.67.

Drinking water with low pH was found to be beneficial for quail and fast-growing poultry in terms of better performance and economic returns [34, 35]. Additionally, low pH levels (pH 5) in drinking water were found to improve the productive and physiological performance of Japanese quails, including increased egg production and average egg weight [36]. The low pH of PAW is due to reactive species like reactive oxygen species and reactive nitrogen species formed when air is ionized in water, producing hydroxyls, nitrates, and nitrites, which generate hydrogen peroxide, nitric acid, and peroxynitrous acid, making PAW acidic [16, 37, 38]. Several previous studies reported that PAW, resulting from water exposure to air plasma for 15 min, had low pH values (2.11 [39], 3.65 [40], 3.66 [37]), like the one used in the current study. PAW doses evaluated in the current study were chosen based on previous studies examining drinking water commercial acidifying agents in quail and broilers, which showed effective results with a pH as low as 4 and a maximum dose of 2 ml per Liter [35, 41].

Non-thermal atmospheric plasma interacting with water results in the generation of PAW. This plasma treatment leads to the nonequilibrium dissociation of water molecules, producing short-lived species such as hydrated electrons and hydroxyl ions. The latter intermediates rapidly react to form stable species, including ozone, superoxides, and hydrogen peroxide. Notably, hydroxyl radicals, with their high reactivity and short lifespan, interact with other liquid components, initiating further reactions in the system [42]. Thus, the marked elevation in serum hydrogen peroxide may be attributed to the saturation of PAW with a diverse range of reactive oxygen and nitrogen species. However, it appears that the production of reactive mediators did not surpass the capability of endogenous antioxidant defences in our experimental model, as indicated by the sustained levels of serum total antioxidant capacity. In a previous scholarly article, lipid peroxidation in chicken wings remained unaffected by the treatment with plasma-activated liquids [43].

Quail total protein primarily comprises albumin and globulins [44, 45]. Globulins are proteins that are mainly involved in the immune defence system. The elevated serum total protein and globulin levels observed in quails receiving PAW-1 supplementation, without affecting the serum albumin level, emphasize the potential of PAW as an immunostimulatory agent [46, 47]. However, this hypothesis was not evaluated in the current study. Supporting our hypothesis, a study by Ahmad et al. [43] mentioned that a higher level of globulin in quail serum is often used as an indicator for measuring immune response. They attributed the improvement in bird immunity to the inhibitory effects of organic acids on the gut system’s microbial population, thereby enhancing non-specific immunity.

The reduction in serum urea and creatinine levels in PAW-treated groups is consistent with the findings observed in gamma-irradiated albino rats [48]. PAW-induced angiogenesis [49] may enhance the capillary filtration coefficient and increase the renal elimination of metabolic waste products. Providing quails with a high-protein diet may result in the development of hyperuricemia and gout [50–52]. Elevated serum uric acid levels associated with a protein-rich diet in poultry can adversely affect kidney function, increase the risk of gout, and negatively impact microbial communities and immunoregulation [53]. Histopathological deterioration in the liver and spleen, haematological abnormalities, and genotoxicity in erythrocytes in layer birds are linked to raised urea levels [54]. Hence, the potential of PAW to eliminate metabolic waste products of protein from circulating blood presents a promising approach to mitigate the inevitable side effects associated with feeding high-protein diets.

Muscle pH is an important post slaughter evaluating factor with effect on meat quality attributes such as meat color, Water holding capacity and the other muscle characteristics [55]. Poultry meats with an ultimate pH between 5.7 and 6.1 are called normal and do not reveal any quality problems [56, 57]. The average pH determined in our study (5.8–6.1) was coherent with the other findings in the literature for quail meat [58, 59].

The colour of food is arguably the most crucial sensory characteristic, being the initial aspect noticed by consumers. It plays a vital role in shaping consumers’ perceptions of a product’s freshness, flavour, and overall quality [60]. The color attributes tend to be strongly associated with the pigment myoglobin, affected by myoglobin oxidation [61, 62]. Several studies have reported that the inclusion of low pH compounds in drinking water has the potential to improve poultry carcass and meat quality characteristics [21, 63, 64]. Similarly, the current study reported improvement in quail carcass quality parameters including increasing meat colour, taste, and overall acceptance score in PAW treated groups. Supporting these finding, Zhao et al. [65] reported that when fresh beef meat was directly treated with PAW, there was an enhancement in the redness index, with no significant alterations observed in the lightness or yellowness indexes. The meat’s redness heightened either because of the oxidizing property of PAW [65] or due to its abundance in nitrite [66].

In our study, quails supplemented with PAW showed an improvement in the taste and overall acceptability of meat. High-voltage PAW has been shown to accelerate the flavour development of food products [67]. It is well known that taste perception is closely associated with textural characteristics experienced during food chewing [68]. The physical effects of PAW, including alterations in pH, oxidation-reduction potential, and the presence of reactive oxygen and nitrogen species, can induce significant changes to the food matrix. These modifications encompass enzyme inactivation, sugar breakdown, and lipid oxidation, thereby directly or indirectly influencing the textural properties of the food matrix [69]. Chicken breast treated with acidic electrolyzed water exhibited superior sensory scores in terms of texture and overall acceptability compared to the control [70]. The current study tracked enhancements in meat colour and taste present opportunities for increased consumer satisfaction and market appeal.

The current study recorded that PAW-2 exhibited a significantly higher *Lactobacilli* count than the control group. Supporting this finding, several studies reported that feed additives with acidic pH, such as organic acids, are utilized as alternatives to antibiotics because it promotes the growth of acid-tolerant beneficial bacteria while inhibiting the growth of acid-labile harmful pathogens [71, 72]. Lactic acid bacteria, like *Lactobacilli*, can flourish in lower pH environments [72, 73] because its high intracellular potassium concentration shields it from acid anions [74, 75]. The elevation of *Lactobacillus spp.* helps in inhibiting the colonization of pathogens such as *Salmonella spp.* in the gastrointestinal tract of broiler chickens [76], leading to improved BW and FCR [77–80]. The decrease in pH levels is associated with an elevated presence of *lactobacilli* populations, as observed in broiler chickens provided with acetic acid in their drinking water and exposed to *Salmonella enteritidis* [76]. Enhanced *Lactobacilli* colonization in the intestines exerts antimicrobial effects on pathogenic microbiota by creating a reduced pH environment in the gut [81]. Additionally, it produces antimicrobial compounds, including volatile fatty acids, organic acids, and bacteriocins [82]. Lactobacilli compete for nutrients, induce modifications in the structure and function of the intestinal epithelium, and flush out pathogens from the gastrointestinal tract, acting as receptor sites to prevent their adherence [83].

Enhanced intestinal colonization of lactobacilli played a significant role in augmenting histomorphometry parameters related to the intestinal absorptive surface, such as villus height [84] and mucosal layer height [81] crypt depth [83]. The observed increase in villus height implies the development of an expanded surface area capable of more efficient absorption of available nutrients [85]. Raised villous height and reduced crypt depth are associated with an enhanced epithelial turnover [86]. The heightened turnover of mucosal cells in the intestinal epithelium of birds plays a role in preserving mucosal integrity in the small intestine, acting as a preventive measure against the entry of pathogenic bacteria [83].

PAW contains a substantial amount of nitrate and nitrite, a powerful relaxant for vascular smooth muscle cells, leading to a reduction in vascular resistance [87]. The acidification of the intestinal environment induced by *lactobacilli* results in the generation of nitric oxide (NO) through non-enzymatic nitrite reduction [88]. Lactobacilli provide additional source for conversion of nitrite to NO [89, 90]. NO acts as a crucial vasorelaxant by activating guanylyl cyclase [91]. All these vasodilator agents might be responsible for the congestion and dilation observed in the hepatic and splenic vasculature especially in PAW-2 group. They also accelerate renal plasma flow and augment glomerular filtration rate [92] and subsequently increase urinary urea and creatinine clearance.

While our study presents promising results, it is important to acknowledge its limitations. This research is preliminary and exploratory, serving as the first work to discuss PAW effects on quail. Thus, we used the minimum number of quail necessary, adhering to the 3R welfare standards (Replacement, Reduction, and Refinement), which emphasize the ethical use and care of animals in research. We were cautious about potential toxic effects. The lack of significant differences in performance could be attributed to the relatively short duration of the experiment (21–42 days) or the limited BW changes occurring during this period. We also investigated potential sex differences within each treatment and found no interaction between sex and treatment, this may be attributed to low number of each sex (3males vs. 3females). Current sample size may be on the lower side, However, the use of multiple replicates within each group helps to mitigate some of these limitations. The findings from our study provide valuable preliminary data. Future research should consider larger sample sizes and extended timelines is necessary to validate our findings and fully understand the mechanisms of PAW.

In conclusion, PAW treatments at doses of 1 ml or 2 ml per Liter of drinking water induced several biochemical, meat quality and histological changes in internal organs including higher *lactobacilli* count and lower serum creatinine and urea levels, as well as improving meat colour, taste, and overall acceptance score. These recorded changes are important for improving health and production in the sustainable quail farming industry. Therefore, further investigations are still needed to fine-tune the appropriate procedure and exact regimen for PAW treatment in quail and other poultry species.

## Materials and methods

All study procedures were approved by the Animal Care and Use Committee of the Faculty of Veterinary Medicine, Assiut University, Egypt (Ethical Approval No. 06/2024/0182).

### Plasma activated water synthesis

PAW syntheses were done at Physics Department, Faculty of Science, Sohag University, (Sohag 82524, Egypt). Procedures details were formerly published by Abd El-Reda et al. [37]. Briefly, PAW was prepared by generating plasma on the distilled water surface using atmospheric air as the working supply gas at room temperature. 100 mL of distilled water was activated by plasma for 15 min, at 28 °C. The physicochemical measurements including pH, electrical conductivity, and hydrogen peroxide and Nitrites of the distilled water after the plasma activation were 3.81 ± 0.06, 150.54 ± 5.97(µS/m), 0.38 ± 0.07(mM/L) and 5.35 ± 2.27(µM/L) respectively.

### Birds and housing

A total of 54 twenty-one-day-old Japanese quail chicks were purchased from a commercial hatchery. Chicks were kept at the same experimental room in multideck batteries, each with four decks and two cages per deck. Each cage, measuring 45 × 50 × 30 cm, accommodated one male and two females, ensuring standardized conditions for the study. A basal diet was formulated to meet the National Research Council (NRC, 1994) recommendations (Table 6) and was offered ad libitum over the experimental period (21-42d). The chicks were fed a starter diet (CP: 24.08%; ME: 2830 kcal) and were kept under routine management practice and an optimum hygienic environment until the end of the experiment. On the first day of the experiment (21 day of age), chicks were randomly assigned to three PAW treatment groups: 0 (Control), 1 ml (PAW-1), and 2 ml (PAW-2) per liter of drinking water. Each treatment group consisted of six replicates (3 birds/cage). Water was provided ad libitum throughout the whole experimental period (21–42 days). The water was supplied via a single water container placed on the upper deck of each battery, connected with plastic tubes leading to nipple drinkers inside each cage. The water was replaced daily with freshly prepared PAW diluted water.

**Table 6:** Ration physicall and chemical composition [93, 94]

	Ingredients	(%)	Chemical composition	(%)
1.	Yellow corn (ground)	49.50	Crude protein (%)	24.08
2.	Soya bean meal	45.00	Metabolizable energy (Mcalkg^− 1^)	2.83
3.	Dried fat	2.20	Calcium (%)	0.87
4.	Ground limestone	1.00	Total phosphorus (%)	0.73
5.	Dicalcium phosphate	1.60	Crude fiber (%)	4.24
6.	Common salt	0.25		
7.	Mineral premix^1^	0.10		
8.	Vitamin premix^2^	0.12		
9.	Methionine	0.13		
10.	Lysine	0.10		
**Total**		**100%**		

^1^Supplied per kg of diet: Fe, 60 mg; Mn, 60 mg; Cu, 6 mg; I, 1 mg; Co, 1 mg; Se, 0.20 mg and Zn, 60 mg

^2^Supplied per kg of diet: Vitamin A, 12,000 IU; vitamin D3, 2200 IU; vitamin E, 26 IU; vitamin K3, 6.25 mg; vitamin B1, 3.75 mg; vitamin B2, 6.6 mg; vitamin B6, 1.5 g; pantothenic acid, 18.8 mg; vitamin B12, 0.31 mg; niacin, 30 mg; folic acid, 1.25 mg; biotin, 0.6 mg and choline chloride, 500 mg

### Birds performance

Live body weight (LBW), average live body weight gain (BWG), feed intake (FI), and feed conversion (FC) were recorded weekly throughout the experimental period (21–42 days).

### Behavioural observations

Direct live observations of quail behavior were conducted three times per week, once daily from 10:00 to 12:00 h, covering 18 cages (i.e., 6 cages per treatment). Instantaneous scan sampling at 10-minute intervals per hour was employed. A trained observer was present in the room during the observation periods but took precautions to minimize disturbance to the birds and maintain a consistent distance 1 m away from the battery cages. The observer was blinded to the treatment. The recorded activities, including drinking, feeding, standing, sitting, preening, stretching, feather pecking, and wall pecking (ethogram in Table 7), were reported for each cage. The data is expressed as the proportion of a certain activity to the total activities recorded/ cage.

**Table 7:** Behavioural ethogram [95–97]

Behaviour	Definition
**Standing**	Posture where both feet are in contact with the floor, and no other body part is in contact with the ground.
**Sitting**	A sitting position is characterized by most of the ventral region of the bird’s body being in contact with the cage floor, with no visible space between the floor and the bird.
**Walking**	Quail walking is observed when the bird is in the process of taking multiple steps, which includes more than two steps
**Feeding**	The quail’s head is positioned inside the feeder, and it is actively pecking at the feed
**Drinking**	The quail’s beak is in contact with the nipples of the drinker, and the bird is actively drinking water
**Grooming**	Preening (Gently pecking or scratching its own feathers), dust bathing, and leg or wing stretching
**Preening**	The bird uses its beak to clean its feathers while either standing or crouching.
**Stretching**	Quail stretching involves the extension of wings and legs, either unilaterally or bilaterally
**Shaking**	The behaviour described as shaking involves the bird moving its head and body while in a standing position
**Feather Peking**	Pecking actions directed towards the feathers of another bird.
**Wall pecking**	The behaviour of pecking at non-edible objects or the ground.

### Blood collection and biochemical parameters

Blood samples were collected from six birds in each group (one bird per cage; 3 males and 3 females) by birds killing using the Islamic slaughter procedures, which involves severing the jugular vein, carotid artery, trachea, and esophagus with a sharp knife in a single swift motion to ensure loss of consciousness and minimize pain [98, 99]. The slaughter method adhered to the Halal protocol established by Malaysian institutes [100], where birds were slaughtered without anesthesia, and the major jugular veins were severed to facilitate effective bleeding [98] on the 42nd day of age. The collected serum samples were stored at − 20 °C until utilized for biochemical parameter analysis.

Total antioxidant capacity and hydrogen peroxide were measured using commercial colorimetric kits (catalog numbers: TA2513 and HP 25, respectively) according to the manufacturer’s instructions (Biodiagnostic, Dokki, Giza, Egypt). Total protein, albumin, aspartate aminotransferase, alanine aminotransferase, urea, and creatinine were all assessed using commercial colorimetric kits (catalog numbers: 310001, 211001, 260001, 264001, 318001, and 234000, respectively) following the manufacturer’s protocol (Egyptian Company for Biotechnology). The serum globulin level was determined by subtracting the serum albumin level from the serum total protein level Agina et al. [44].

### Carcass quality measurements

Following blood collection, the birds were subjected to scalding in a water tank at 55–60 °C for 30 s, followed by plucking and evisceration, including the removal of the vent with intestines. The individual weights of the empty carcass, heart, liver, gizzard, and spleen were recorded and expressed as a percentage of body weight [101].

Water-holding capacity: The method described by [102] was employed to assess the water-holding capacity of post-rigor chilled quail breast and leg muscles. Approximately 300 mg of the sample was compressed between two filter papers (70mmu) and ceramic plates, applying 5 kg weight for 3 min. After pressing, the compressed sample was carefully separated from the moist filter paper, and its weight was immediately measured. The released water (RW) percentage was calculated using the formula: RW% = (W1 - W2)/W1 * 100, where W1 represents the weight of the meat sample, and W2 is the weight of the meat sample after pressing. Water-holding capacity was expressed as the percentage of water retained by the meat sample, calculated as: Water-holding capacity (% water retained) = 100 – RW%.

Sensory assessment: Consumer-based sensory panels were conducted to evaluate the acceptability of quail breast and thigh meat from quails subjected to different dietary treatments. Post-rigor frozen quail breasts and thighs were boiled until completely cooked (~ 10–15 min), cut into parts, and kept warm (60–70 °C) in Petri dishes for panelist evaluation. Three members of the Food Hygiene Department at the Faculty of Veterinary Medicine, Assiut University, conducted blinded sensory evaluations, ensuring unbiased results free from prior knowledge influence. Sample order was randomized to mitigate sampling order bias. Panelists rated cooked quail breast and thigh samples for colour, taste, juiciness, chewiness, and flavour using a 9-point hedonic scale, where 1 = dislike extremely, 5 = neither like nor dislike, and 9 = like extremely ( [103]). Overall acceptability was determined as the mean of the evaluated traits.

Determination of pH [104]: Five grams of quail breast or thigh muscles were added to 45 ml of distilled water at 25 °C in a stomacher (Seward 400) bag and subjected to stomaching for 2 min. The mixture was then left for 10 min at room temperature. A pH meter (SD 50 pH meter, Germany), calibrated with buffers at pH 7 and 4, was immersed into the solution to determine the pH of the sample.

### Intestinal microbial analysis

The gastrointestinal tracts of euthanized quails were gathered for microbiological examination. Aseptically, 1 gram of intestinal contents was collected from each quail (one bird per cage) for the enumeration of total bacterial count and Lactobacillus. These samples were meticulously stored in cryovials at -80 °C until analysis. Microbial miniaturized plating was conducted following a previously published method [105]. Briefly, the intestinal contents (1 g) of each sample were thawed at room temperature for 15 min and were serially diluted (10-fold) with physiological saline in a 96-well plate. Ten microliters of each dilution were plated on Standard Plate Count Agar (Lab M Limited, Lancashire, UK) for the total bacterial count and MRS agar plates (Lab M Limited, Lancashire, UK) for the lactobacilli count. Total bacterial counts were incubated aerobically at 37 °C for 24 h, while lactobacilli counts were incubated anaerobically at 37 °C for 48 h using a Gas-Pak anaerobic system (Becton Dickinson Microbiology Systems). After incubation, colonies were counted and recorded as colony-forming units per gram (cfu/g) of the sample, expressed as log10 cfu/g. The average of the counts from two plates was calculated.

### Histopathology

The intestine, liver, and spleen tissue samples were dissected and promptly fixed in 10% formalin. Following dehydration with increasing concentrations of ethyl alcohol (70–100%) and two passes in xylol, the samples were embedded in paraffin and sectioned at 5 μm using a rotary microtome. Subsequently, the histological slides underwent staining with hematoxylin and eosin (H&E) following the method described by [106].

Standard light microscopy was utilized for the evaluation of the histological sections. Quantitative histomorphometry measurements, specifically intestinal villus lengths and areas, were conducted using the NIH ImageJ software [107]. Measurements were taken from photomicrographs captured at 40× magnification. The lengths of both the villi and crypt regions were measured for a total of six selected individual and apparently complete, full-sized intestinal villi that exhibited no bending or mechanical damage in each sample. All measurements were performed by a single individual who was blinded to the treatments to ensure unbiased data recording. Crypt to villus ratios were then calculated using the length data, as described by [108].

### Statistical analysis

The cage was designated as the experimental unit for analysis. Data were subjected to one-way analysis of variance using the General Linear Models (GLM) procedure in SPSS, version 16. Post hoc comparisons were conducted using Tukey’s test upon detecting significant differences. Statistical significance was established at *P* < 0.05.

## Data Availability

The datasets used and/or analysed during the current study are available from the corresponding author on reasonable request.
